# Exploring the genetic and epigenetic origins of juvenile myelomonocytic leukemia using newborn screening samples

**DOI:** 10.1038/s41375-021-01331-0

**Published:** 2021-06-28

**Authors:** Astrid Behnert, Julia Meyer, Jahan-Yar Parsa, Aaron Hechmer, Mignon L. Loh, Adam Olshen, Adam J. de Smith, Elliot Stieglitz

**Affiliations:** 1 Department of Pediatrics, Benioff Children’s Hospital, University of California, San Francisco, San Francisco, CA USA; 2 Tecan Genomics Inc, Redwood City, CA USA; 3 Department of Epidemiology and Biostatistics, University of California, San Francisco, San Francisco, CA USA; 4 Helen Diller Family Comprehensive Cancer Center, University of California, San Francisco, San Francisco, CA USA; 5 Center for Genetic Epidemiology, University of Southern California, Los Angeles, CA USA

**Keywords:** Genetics research, Cancer epigenetics

## To the Editor:

Juvenile myelomonocytic leukemia (JMML) is a rare and frequently fatal myeloproliferative neoplasm of early childhood, with a median age of diagnosis of approximately 2 years [1]. The disease is characterized by splenomegaly, thrombocytopenia, peripheral monocytosis and elevated hemoglobin F [1]. Allogeneic hematopoietic stem cell transplantation (HSCT) is the only curative therapy; however, five-year event-free (EFS) and overall survival (OS) remains poor, typically due to relapse [2]. Interestingly, in rare cases, patients experience spontaneous resolution without the need for extensive therapy [3, 4], highlighting the need for a better understanding of this enigmatic disease.

Children with RASopathies including Neurofibromatosis type 1 and CBL syndrome are at risk of acquiring JMML if they develop loss of heterozygosity in their bone marrow compartment for *NF1* and *CBL*, respectively [4, 5]. Nearly 95% of JMML patients have somatic or germline mutations in genes encoding proteins that activate the RAS/MAPK pathway (*NF1, CBL, NRAS, KRAS, RRAS, RRAS2, FLT3, PTPN11*) [1, 4]. Several groups have documented secondary mutations at diagnosis that occur in one-third of patients [6, 7]. These mutations include alterations in epigenetic regulating genes, transcription factors, the spliceosome complex and signal transduction pathways. While the canonical RAS pathway mutations lack prognostic significance, secondary mutations are associated with a poor prognosis [6]. Considering the role of epigenetics in other cancers and the fact that DNA methylation plays a critical role in the differentiation of both fetal and adult hematopoietic stem cells [8], our research interest has focused on DNA methylation in JMML. We and others have recently shown that DNA methylation profiles are predictive of outcome [9–12]. Patients who experience spontaneous resolution have a methylation pattern similar to healthy, age-appropriate controls, while hypermethylated DNA signatures are common in patients who have more aggressive disease [9–11]. In our experience, altered DNA methylation is tied to the presence of secondary mutations, as all patients who present with more than one genetic mutation have hypermethylated DNA profiles. Intriguingly, only a small proportion of these mutations occur in genes that are known to regulate DNA methylation [6]. We therefore carried out experiments to investigate whether aberrant DNA methylation drives leukemogenesis, leading to the acquisition of additional mutations, or whether aberrant methylation is a secondary event to primary RAS pathway mutations.

## Methods

### Study subjects

We identified 35 patients that were born in the state of California from 1990 to 2017 who were confirmed to have JMML per World Health Organization criteria [1] and were previously consented to participate in a JMML tissue bank study. None of the patients had Noonan syndrome. Diagnostic JMML material was available for all 35 of these patients. Guthrie cards from the 35 patients were obtained from the California Department of Public Health California Biobank Program. In addition, Guthrie cards were obtained from the California Biobank Program for 12 healthy control subjects born in California who did not develop cancer during childhood. DNA was extracted using standard methods and targeted deep sequencing (Supplemental Table 1) as well as methylation profiling were performed. For additional details, see Supplemental Methods.

## Results

### Genetic changes in newborn blood samples

We analyzed a total of 35 newborn blood screening (NBS) cards from children who developed JMML later in childhood and from 12 healthy controls. Clinical characteristics at disease onset showed a median age at diagnosis of 1.5 years, elevated white blood cell count (WBC), monocytes and hemoglobin F (Supplementary Tables 2 and  3). At diagnosis of JMML, somatic mutations were identified in 34 of 35 patients. The most common mutations were in *PTPN11* (12), *NRAS* (7) and *KRAS* (7) (Supplementary Fig. 1A). Ten of the 34 patients had a secondary mutation at diagnosis. The most common secondary mutations were in *NF1* (2) and *SETBP1* (2). Of the 34 patients who had a somatic mutation present upon diagnosis of JMML, those mutations were found in newborn cards in 13 (38%) patients using a VAF cut-off of 0.01. Clonal mutations (VAF > 15%) were found in 9 of the 34 (26%) patients. Somatic mutations in *NRAS* (4) and *PTPN11* (3) were the most common detected at birth (Supplementary Fig. 1B). In three patients with germline *CBL* mutations, loss of heterozygosity (LOH) was detected at diagnosis but not at birth. Of the 10 patients who had a secondary mutation at diagnosis, none of those were found in newborn blood samples. Patients who had a somatic mutation detected at birth were significantly younger at diagnosis with a median age of 7.1 months compared to 19.8 months in patients who had no mutations at birth (p = 0.03) (Supplemental Table 4). However, no difference was observed in EFS or OS for patients with or without somatic mutations at birth (Supplementary Fig. 2). Targeted deep sequencing did not identify any mutations in the 12 healthy controls included in our study.

### Methylation profiling of newborn blood samples

**Fig. 1:**
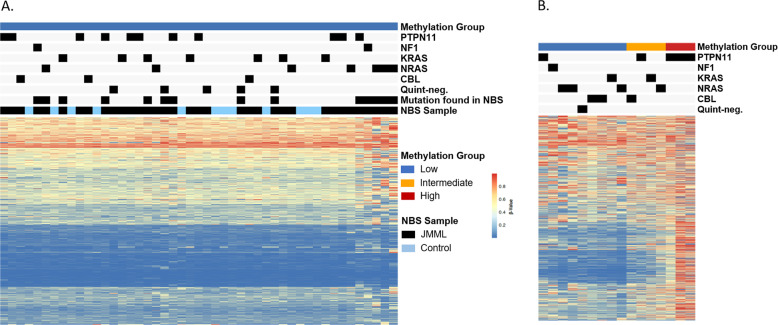
Newborn blood spot cards display a low DNA methylation signature. **A** JMML NBS or control NBS samples profiled by MethylSeq classified according to the international JMML methylation consensus signature [12]. **B** Methylation status at diagnosis of patients (*n* = 16) reported in this study. *Panel A and B* Heatmaps show the beta values of 1386 CpG loci used for methylation classification.

To better understand when DNA methylation changes occur in patients, we profiled all 35 patient and 12 control NBS cards with a custom-capture targeted MethylSeq assay. Minimum distance to the nearest centroid classified all NBS cards as having “low” DNA methylation using the international, consensus definition [12] (Fig. 1A). While all NBS cards were categorized as displaying a low methylation (LM) signature, three patients clustered separately from all other samples (Supplementary Fig. 3). Notably, all three of these patients had a clonal *NRAS* mutation detected at birth.

### Comparison of methylation profiles at birth and at diagnosis

**Fig. 2:**
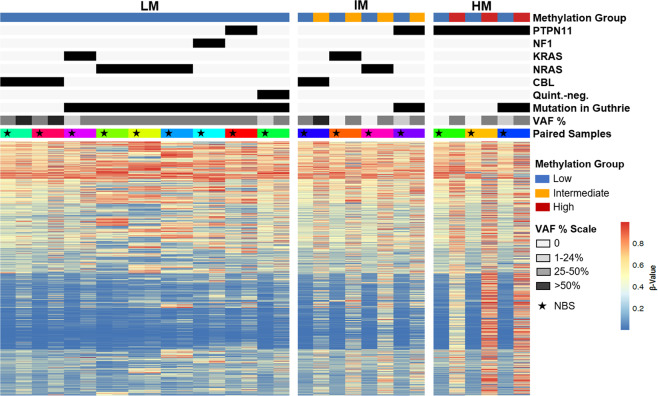
Genetic mutations precede changes in DNA methylation. Genetic and methylation profiling of paired NBS and diagnostic samples from JMML patients are displayed. The star indicates the NBS sample of the pair.

Of the 35 patients, 16 had sufficient DNA available from diagnosis for DNA methylation analysis (Fig. 2). These samples were classified as LM, intermediate (IM) or high methylation (HM). Nine patients had a LM signature at diagnosis, 4 were IM and 3 were HM (Figs. 1B and 2). One patient, UPN3153, had serial samples permitting longitudinal profiling at birth, diagnosis, and post-chemotherapy. The allelic frequency of the patient’s *KRAS* mutation was 0% at birth, 39% at diagnosis and 27% post-chemotherapy. The NBS card was designated LM while both the diagnostic and post-chemotherapy timepoints were IM (Supplemental Figure 4). Despite detection of mutations in nine of the corresponding NBS cards, of which seven were present at a VAF ≥ 19%, all patients had lower methylation at birth compared to diagnosis of JMML (Fig. 2).

## Discussion

In this study, we used next generation sequencing to detect genetic mutations and DNA methylation status to determine the sequence of events that lead to the development of JMML.

We identified somatic RAS pathway mutations in 13/34 (38%) of children that developed JMML later in their life, confirming the prenatal origin of this malignancy in nearly half of patients. We found that patients who had a somatic mutation detected at birth were significantly younger at diagnosis compared to patients who had no mutations at birth. This is consistent with a previous report by Matsuda et al who reported a cohort of seven JMML patients who had an earlier onset of disease when a mutation was present at birth [13]. Interestingly, only 2 of 7 patients with *KRAS* mutations at diagnosis were detected in the NBS cards. This is somewhat surprising considering that patients with *KRAS* mutations typically present at a younger age compared to those with clinical NF1 [1]. Of the two patients with NF1 in our cohort, both had somatic secondary hits in their JMML samples. UPN1070 presented at 7 months and was found to have a second *NF1* mutation and UPN0969 presented at 77 months and was found to have loss of heterozygosity due to uniparental disomy (Supplemental Table 2). UPN1070 had a somatic alteration in NF1 detected in the NBS card at birth, while UPN0969 did not.

A report by Gale et al demonstrated that genomic fusion sequences are present and detectable in neonatal blood spots of patients who went on to develop *MLL* rearranged infant leukemia [14], illustrating that preleukemic clones can be detected at birth in patients that present with leukemia early in life. In children with B-precursor ALL, a high frequency of leukemic clones has been observed in NBS samples across cytogenetic subtypes, including *ETV6*-*RUNX1* fusion [15] and high hyperdiploid ALL [16]. However, a wide variation in latency to the time of diagnosis of leukemia, as observed for some JMML patients in our study, suggests that postnatal factors also play an important role in disease progression [17].

Methylation profiling of NBS cards from children in our study who went on to develop JMML revealed methylation signatures that were similar to normal age-matched controls. Several patients had diagnostic leukemia that was classified as intermediate or high methylation. However, all newborn cards were classified as low methylation at birth using the recent international consensus definition [12] including patients who had clonal mutations in their NBS card. Therefore, we conclude that aberrant DNA methylation is a secondary event to genetic changes.

The causes of somatic mutations at birth and the subsequent alteration in DNA methylation are still unknown. One possibility is that prenatal environmental exposures can cause mutations or changes in DNA methylation as has been hypothesized in the development of childhood ALL [18]. However, we did not collect data about prenatal environmental exposures for JMML patients.

In summary, our findings demonstrate that somatic mutations occur *in utero* in nearly half of JMML patients and that genetic alterations precede aberrant DNA methylation. Further investigation is required to explore the potential mechanisms of altered DNA methylation in this disease.

## Supplementary information

Supplemental Methods

Supplemental Table 1

Supplemental Table 2

Supplemental Table 3

Supplemental Table 4

Supplemental Figure Legends

Supplemental Figure 1

Supplemental Figure 2

Supplemental Figure 3

Supplemental Figure 4

## Data Availability

The datasets generated for this study are available in the Synapse repository, 10.7303/syn25834831.

## References

[1] Locatelli F, Niemeyer CM (2018). How I treat juvenile myelomonocytic leukemia. Blood.

[2] Locatelli F, Nöllke P, Zecca M, Korthof E, Lanino E, Peters C (2005). Hematopoietic stem cell transplantation (HSCT) in children with juvenile myelomonocytic leukemia (JMML): Results of the EWOG-MDS/EBMT trial. Blood.

[3] Matsuda K, Shimada A, Yoshida N, Ogawa A, Watanabe A, Yajima S (2007). Spontaneous improvement of hematologic abnormalities in patients having juvenile myelomonocytic leukemia with specific RAS mutations. Blood.

[4] Loh ML, Sakai DS, Flotho C, Kang M, Fliegauf M, Archambeault S, et al. Mutations in CBL occur frequently in juvenile myelomonocytic leukemia. Blood. 2009;114:1859–63. 10.1182/blood-2009-01-198416 PMC2738571 19571318

[5] Steinemann D, Arning L, Praulich I, Stuhrmann M, Hasle H, Stary J (2010). Mitotic recombination and compound-heterozygous mutations are predominant NF1-inactivating mechanisms in children with juvenile myelomonocytic leukemia and neurofibromatosis type 1. Haematologica.

[6] Stieglitz E, Taylor-Weiner AN, Chang TY, Gelston LC, Wang Y-DD, Mazor T (2015). The genomic landscape of Juvenile myelomonocytic leukemia. Nat Genet.

[7] Sakaguchi H, Okuno Y, Muramatsu H, Yoshida K, Shiraishi Y, Takahashi M (2013). Exome sequencing identifies secondary mutations of SETBP1 and JAK3 in juvenile myelomonocytic leukemia. Nat Genet.

[8] Lipka DB, Wang Q, Cabezas-Wallscheid N, Klimmeck D, Weichenhan D, Herrmann C (2014). Identification of DNA methylation changes at cis-regulatory elements during early steps of HSC differentiation using tagmentation-based whole genome bisulfite sequencing. Cell Cycle.

[9] Stieglitz E, Mazor T, Olshen AB, Geng H, Gelston LC, Akutagawa J (2017). Genome-wide DNA methylation is predictive of outcome in juvenile myelomonocytic leukemia. Nat Commun.

[10] Lipka DB, Witte T, Toth R, Yang J, Wiesenfarth M, Nöllke P *et al*. RAS-pathway mutation patterns define epigenetic subclasses in juvenile myelomonocytic leukemia. Nat Commun. 2017; 8. 10.1038/s41467-017-02177-w. 10.1038/s41467-017-02177-w PMC5736667 29259247

[11] Murakami N, Okuno Y, Yoshida K, Shiraishi Y, Nagae G, Suzuki K (2018). Integrated molecular profiling of juvenile myelomonocytic leukemia. Blood.

[12] Schoenung M, Meyer J, Noellke P, Olshen A, Hartmann M, Murakami N *et al*. International consensus definition of DNA methylation subgroups in juvenile myelomonocytic leukemia. Clin Cancer Res Press 2020. 10.1158/1078-0432.CCR-20-3184 PMC7785676 33139265

[13] Matsuda K, Sakashita K, Taira C, Tanaka-Yanagisawa M, Yanagisawa R, Shiohara M (2010). Quantitative assessment of PTPN11 or RAS mutations at the neonatal period and during the clinical course in patients with juvenile myelomonocytic leukaemia. Br J Haematol.

[14] Gale KB, Ford AM, Repp R, Borkhardt A, Keller C, Eden OB (1997). Backtracking leukemia to birth: Identification of clonotypic gene fusion sequences in neonatal blood spots. Proc Natl Acad Sci USA.

[15] Wiemels JL, Xiao Z, Buffler PA, Maia AT, Ma X, Dicks BM (2002). In utero origin of t(8;21) AML1-ETO translocations in childhood acute myeloid leukemia. Blood.

[16] Panzer-Grümayer ER, Fasching K, Panzer S, Hettinger K, Schmitt K, Stöckler-Ipsiroglu S (2002). Nondisjunction of chromosomes leading to hyperdiploid childhood B-cell precursor acute lymphoblastic leukemia is an early event during leukemogenesis. Blood.

[17] Taub JW, Konrad MA, Ge Y, Naber JM, Scott JS, Matherly LH (2002). High frequency of leukemic clones in newborn screening blood samples of children with B-precursor acute lymphoblastic leukemia. Blood.

[18] Timms JA, Relton CL, Rankin J, Strathdee G, McKay JA (2016). DNA methylation as a potential mediator of environmental risks in the development of childhood acute lymphoblastic leukemia. Epigenomics.

