# Supplementary material for: Exploring the genetic and epigenetic origins of juvenile myelomonocytic leukemia using newborn screening samples
Source: Leukemia. 2021 Jun 28;36(1):279–82. doi: 10.1038/s41375-021-01331-0 (PMC8720242; doi:10.1038/s41375-021-01331-0)
Supplement: Supplementary file 1 — Supplemental Methods [file 41375_2021_1331_MOESM1_ESM.docx]

***Supplemental Methods***

*DNA extraction*

Genomic DNA from Guthrie cards and from peripheral blood or bone marrow at the time of diagnosis of JMML were extracted using DNeasy blood and tissue kits (Qiagen, Hilden, Germany). Quantity and integrity of the extracted DNA was measured using Qubit (Thermo Fisher Scientific, Waltham, MA).

*Targeted deep sequencing*

DNA samples were sequenced using a custom amplicon-based targeted sequencing approach. Libraries were prepared (Paragon Genomics, Hayward, CA) using 20ng (for Guthrie cards) or 10ng (for diagnosis samples) of genomic DNA per primer pool targeting 26 genes in total that are recurrently mutated in JMML (Supplemental Table 1). Libraries were assessed by Bioanalyzer (Agilent, Santa Clara, CA) and sequenced on a HiSeq 4000 (Illumina, San Diego, CA). FASTQ reads were trimmed with fastp to remove amplicon primer sequences and adapters then aligned using BWA^5,^. Variants called with VarScan2^3^. Total mean and median coverage per alteration site examined in BAM files was 39,500× and 38,000x respectively. Minimum depth of ≥ 100 reads and ≥ 10 alternate reads were required to call alterations. Mean and median unique reads per mutation was 6,600x and 6,400x respectively. Minimum variant allele fraction (VAF) of 0.01 was required for reporting.

*Methylation analysis*

Extracted genomic DNA was treated with RNaseA (10mg/ml) for 30mins at 37°C and then immediately processed using the TrueMethyl oxBS Module according to protocol (Tecan Genomics Inc., Redwood City, CA.). Converted ssDNA was quantified using the Qubit ssDNA assay (Thermo Fisher Scientific, Waltham, MA) and 100ng used as input for a 3000 CpG loci custom-made Targeted MethylSeq assay (Tecan Genomics Inc., Redwood City, CA). Final libraries were quality checked using the Bioanalyzer High Sensitivity DNA Analysis kit (Agilent Technologies, Santa Clara, CA) and quantified using the Qubit HS assay (Thermo Fisher Scientific, Waltham, MA) before pooling for next generation sequencing. Single-index library pools were sequenced on the Illumina HiSeq 4000 using paired-end 150bp reads.

Sequence reads were trimmed using Cutadapt^4^ to remove adapters and methylation status called using Bismark^5^. Those CpGs that had ≤50x median coverage were removed from downstream analysis, resulting in 84 CpG sites removed. Samples were classified into one of three methylation subgroups – low, intermediate, or high – using 1386 probes identified in an international project to establish a consensus definition of DNA methylation subgroups in JMML^6^. Minimum distance to the centroid was used to determine methylation classification for Guthrie samples. Hclust in R (version 3.6.3) using Ward’s method was used for hierarchical clustering of samples.

*Statistical analysis*

The median follow up among those who survived was 3.7 years (range 0.4-23.7 years). Overall survival (OS) was defined as time from initial diagnosis to death resulting from any cause. Event-free survival (EFS) was defined as occurrence of relapse or death from any cause^6^. Both were estimated using the Kaplan-Meier method. Statistically significant differences between somatic mutations being present at birth were tested using Fisher’s exact test for categorical variables and the Mann-Whitney U test for continuous variables. The significance cutoff throughout was p < 0.05. All calculations were performed using GraphPad Prism software (v8.0).

*Data Availability*

The datasets generated for this study are available in the Synapse repository, DOI: 10.7303/syn25834831.

*Institutional Review Board*

The study was designed in accordance with the Declaration of Helsinki and reviewed and approved by the institutional review board at UCSF and the Committee for the Protection of Human Subjects in the State of California.

***Supplemental References***

1. Chen S, Zhou Y, Chen Y, Gu J. fastp: an ultra-fast all-in-one FASTQ preprocessor. *Bioinformatics* 2018; **34**: i884–i890.

2 Li H, Durbin R. Fast and accurate short read alignment with Burrows-Wheeler transform. *Bioinformatics* 2009; **25**: 1754–1760.

3 Koboldt DC, Zhang Q, Larson DE, Shen D, McLellan MD, Lin L *et al.* VarScan 2: somatic mutation and copy number alteration discovery in cancer by exome sequencing. *Genome Res* 2012; **22**: 568–576.

4 Martin M. Cutadapt removes adapter sequences from high-throughput sequencing reads. *EMBnet.journal; Vol 17, No 1 Next Gener Seq Data Anal - 1014806/ej171200*  2011.http://journal.embnet.org/index.php/embnetjournal/article/view/200.

5 Krueger F, Andrews SR. Bismark: a flexible aligner and methylation caller for Bisulfite-Seq applications. *Bioinformatics* 2011; **27**: 1571–1572.

6 Schoenung M, Meyer J, Noellke P, Olshen A, Hartmann M, Murakami N *et al.* International consensus definition of DNA methylation subgroups in juvenile myelomonocytic leukemia. *Clin Cancer Res Press* 2020.
