# Supplementary material for: Exploring the genetic and epigenetic origins of juvenile myelomonocytic leukemia using newborn screening samples
Source: Leukemia. 2021 Jun 28;36(1):279–82. doi: 10.1038/s41375-021-01331-0 (PMC8720242; doi:10.1038/s41375-021-01331-0)
Supplement: Supplementary file 2 — Supplemental Table 1 [file 41375_2021_1331_MOESM2_ESM.docx]

***Supplemental Table 1: Targeted Sequencing Panel***

| **Gene** |
| --- |
| *ASXL1* |
| *BRAF* |
| *CBL* |
| *DNMT3A* |
| *ETV6* |
| *EZH2* |
| *FLT3* |
| *GATA2* |
| *JAK3* |
| *KRAS* |
| *MAP2K1* |
| *NF1* |
| *NRAS* |
| *PTPN11* |
| *RAC2* |
| *RAF1* |
| *RIT1* |
| *RRAS* |
| *RRAS2* |
| *RUNX1* |
| *SAMD9* |
| *SAMD9L* |
| *SETBP1* |
| *SH2B3* |
| *SOS1* |
| *ZRSR2* |
