# Supplementary material for: Exploring the genetic and epigenetic origins of juvenile myelomonocytic leukemia using newborn screening samples
Source: Leukemia. 2021 Jun 28;36(1):279–82. doi: 10.1038/s41375-021-01331-0 (PMC8720242; doi:10.1038/s41375-021-01331-0)
Supplement: Supplementary file 3 — Supplemental Table 2 [file 41375_2021_1331_MOESM3_ESM.docx]

***Supplemental Table 2: Clinical Patient Characteristics at Diagnosis***

| Characteristics | Whole Cohort (n=35) | Data n/a |
| --- | --- | --- |
| *Gender, male, n (%)* | 23 (65.71%) |  |
| *Median patient age at diagnosis, months (range)* | 17.6 (2.5 - 91.6) |  |
| *Mean WBC count at diagnosis, x10^9^/L (range)* | 50.28 (4.00 - 366.70) | n = 2 |
| *Mean monocyte count at diagnosis, x10^9^/L (range)* | 349.70 (0.40 - 4450) | n = 4 |
| *Mean platelet count at diagnosis, x10^9^/L (range)* | 41.50 (5.00 - 124.00) | n = 2 |
| *HbF elevated for age, n (%)* | 14 (38.89%) | n = 6 |
| *Karyotype* |  | n = 3 |
| *abnormal, n (%)* | 11 (31.43%) |  |
| *Monosomy 7, n (%)* | 5 (14.29%) |  |
| *Secondary mutations at diagnosis, n (%)* | 10 (28.57%) |  |
