# Supplementary material for: Exploring the genetic and epigenetic origins of juvenile myelomonocytic leukemia using newborn screening samples
Source: Leukemia. 2021 Jun 28;36(1):279–82. doi: 10.1038/s41375-021-01331-0 (PMC8720242; doi:10.1038/s41375-021-01331-0)
Supplement: Supplementary file 4 — Supplemental Table 3 [file 41375_2021_1331_MOESM4_ESM.docx]

***Supplemental Table 3: Individual Patient Characteristics***

| UPN | Primary Mutation  at Diagnosis | Germline/somatic? | Primary Mutation VAF at Diagnosis | Primary Mutation VAF at Birth | Primary Mutation Coverage Guthrie Card | Secondary Mutation at Diagnosis | Secondary Mutation VAF at Diagnosis | Secondary Mutation VAF at Birth | Secondary Mutation Coverage Guthrie Card | Methylation Status at Diagnosis | Monosomy 7 Status at Diagnosis | Age at Diagnosis (months) | OS | EFS | Time to Relapse (months) |
| --- | --- | --- | --- | --- | --- | --- | --- | --- | --- | --- | --- | --- | --- | --- | --- |
| UPN0585 | *NRAS* p.G13D | somatic | 0.32 | 0 | 25478 | *SETBP1* p.D868G | 0.32 | 0 | 18330 | IM | No | 17.6 | No | No | 8.5 |
| UPN0868 | *PTPN11* p.E76K | somatic | 0.28 | 0 | 68760 |  |  |  |  | n/a | No | 10.4 | Yes | Yes |  |
| UPN0906 | *PTPN11* p.E76K | somatic | 0.57 | 0 | 57940 | *ASXL1* p.E727* | 0.35 | 0 | 49961 | n/a | No | 54.9 | Yes | No | 35.6 |
| UPN0969 | *NF1* p.T1972fs | somatic | 0.89 | 0.5 | 37094 | *NRAS* p.G12V | 0.4 | 0 | 27033 | n/a | No | 77.1 | No | No | 4.8 |
| UPN1043 | *PTPN11* p.E76K | somatic | 0.46 | 0 | 55827 |  |  |  |  | n/a | No | 41.8 | No | No | 18.4 |
| UPN1070 | *NF1 p.Q1447*; NF1* p.I679fs | germline; somatic | 0.49; 0.43 | 0.5; 0.28 | 53888; 37500 |  |  |  |  | LM | n/a | 7.6 | n/a | n/a | n/a |
| UPN1333 | *CBL* p.Y371H | germline | 0.76 | 0.49 | 39259 |  |  |  |  | IM | No | 7.2 | No | No | 13.0 |
| UPN1447 | *PTPN11* p.E76K | somatic | 0.3 | 0 | 64159 | *DNMT3A* p.G707fs | 0.16 | 0 | 41466 | HM | No | 31.2 | No | No | 5.6 |
| UPN1669 | *NRAS* p.G12D | somatic | 0.52 | 0.41 | 24811 |  |  |  |  | LM | No | 3.1 | Yes | No | 17.8 |
| UPN1740 | *NRAS* p.Q61K | somatic | 0.43 | 0 | 43711 |  |  |  |  | n/a | No | 30.1 | No | No |  |
| UPN1826 | *NRAS* p.G12D | somatic | sanger sequencing | 0.46 | 29632 |  |  |  |  | LM | No | 2.5 | Yes | Yes |  |
| UPN1897 | *PTPN11* p.G503V | somatic | 0.68 | 0 | 43830 | *NF1* p.I679fs | 0.14 | 0 | 33000 | n/a | No | 64.0 | No | No | 5.6 |
| UPN1970 | *SH2B3* p.E400K | germline | 0.42 | 0.48 | 12274 |  |  |  |  | n/a | No | 7.0 | Yes | Yes |  |
| UPN2357 | *CBL* p.Q409R | germline | 0.91 | 0.5 | 11993 |  |  |  |  | LM | No | 8.4 | Yes | Yes |  |
| UPN2515 | *CBL* c.1096-4_1096-1delAAAG | germline | 0.75 | 0.4 | 7049 |  |  |  |  | LM | n/a | 20.7 | Yes | Yes |  |
| UPN2531 | *PTPN11* p.E69K | somatic | 0.39 | 0.09 | 67675 | *SH2B3* p.W262* | 0.35 | 0 | 46168 | HM | No | 35.5 | No | No | 5.5 |
| UPN2609 | *KRAS* p.G12D | somatic | 0.32 | 0 | 31736 | *KRAS* p.T58I | 0.05 | 0 | 27168 | n/a | n/a | 19.8 | n/a | n/a | n/a |
| UPN2629 | *KRAS* p.G12V | somatic | 0.17 | 0 | 33061 |  |  |  |  | n/a | No | 19.2 | Yes | Yes |  |
| UPN2682 | *PTPN11* p.G60R | somatic | 0.46 | 0.19 | 48144 |  |  |  |  | IM | No | 17.9 | n/a | n/a | n/a |
| UPN2751 | *PTPN11* p.E76K | somatic | 0.47 | 0.29 | 73246 |  |  |  |  | LM | No | 7.1 | No | n/a | n/a |
| UPN2857 | *NRAS* p.Q61L | somatic | 0.37 | 0 | 41758 |  |  |  |  | n/a | No | 12.3 | Yes | Yes |  |
| UPN2860 | *KRAS* p.G13D | somatic | 0.16 | 0 | 38513 |  |  |  |  | n/a | Yes | 7.6 | Yes | Yes |  |
| UPN2917 | *KRAS* p.G13D | somatic | sanger sequencing | 0.021 | 37226 |  |  |  |  | n/a | No | 11.3 | Yes | Yes |  |
| UPN2937 | *NRAS* p.G13D | somatic | 0.84 | 0.012 | 27416 | *SETBP1*  p.D868G | 0.45 | 0 | 15059 | n/a | Yes | 91.6 | Yes | Yes |  |
| UPN2964 | *FLT3* fusion | somatic | 0.3 | 0.25 | present |  |  |  |  | LM | Yes | 4.6 | Yes | Yes |  |
| UPN2983 | *KRAS* p.G13D | somatic | 0.42 | 0 | 38741 |  |  |  |  | n/a | No | 11.8 | Yes | Yes |  |
| UPN3054 | *KRAS* p.G12D | somatic | 0.3 | 0.016 | 19093 |  |  |  |  | LM | No | 3.4 | Yes | Yes |  |
| UPN3062 | none | n/a |  |  |  |  |  |  |  | n/a | No | 46.3 | Yes | Yes |  |
| UPN3063 | *PTPN11* p.E76K | somatic | 0.35 | 0 | 65988 |  |  |  |  | n/a | No | 28.4 | No | No | 11.9 |
| UPN3064 | *PTPN11* p.D61Y | somatic | 0.42 | 0 | 43689 | *JAK3* p.R657Q | 0.23 | 0 | 21606 | n/a | Yes | 33.7 | No | No | 8.3 |
| UPN3065 | *JAK3 p.R887H; FLT3* p.D835H | germline; somatic | 0.47; 0.38 | 0.48; 0.3 | 10783; 28739 |  |  |  |  | n/a | No | 5.3 | Yes | Yes |  |
| UPN3066 | *NRAS* p.G13D | somatic | 0.41 | 0.39 | 28137 |  |  |  |  | LM | Yes | 5.3 | Yes | Yes |  |
| UPN3067 | *PTPN11* p.E76K | somatic | 0.46 | 0 | 70027 |  |  |  |  | n/a | No | 35.2 | Yes | Yes |  |
| UPN3153 | *KRAS* p.G13D | somatic | 0.39 | 0 | 20454 |  |  |  |  | IM | No | 17.6 | Yes | Yes |  |
| UPN3168 | *PTPN11* p.G60V | somatic | 0.47 | 0 | 27883 | *NF1* p. Y794* | 0.08 | 0 | 25109 | HM | No | 50.8 | Yes | No | n/a |
