# Supplementary material for: Exploring the genetic and epigenetic origins of juvenile myelomonocytic leukemia using newborn screening samples
Source: Leukemia. 2021 Jun 28;36(1):279–82. doi: 10.1038/s41375-021-01331-0 (PMC8720242; doi:10.1038/s41375-021-01331-0)
Supplement: Supplementary file 5 — Supplemental Table 4 [file 41375_2021_1331_MOESM5_ESM.docx]

**Supplemental Table 4: Univariate Analysis Based on Alteration Status at Birth**

| Variable | Somatic alteration at birth (n = 13) | No somatic alteration at birth (n = 21) | p value |
| --- | --- | --- | --- |
| *Gender, male, n (%)* | 8 (61.54%) | 15 (71.43%) | 0.54 |
| *Median patient age at diagnosis, months (range)* | 7.1 (2.5 - 91.6) | 19.77 (7.0 - 64.0) | 0.03 |
| *Mean WBC count at diagnosis, x10^9^/L (range)* | 57.23 (9.20 - 181.00) | 46.10 (4.00 - 366.70) | 0.66 |
| *Mean platelet count at diagnosis, x10^9^/L (range)* | 42.17 (10.00 - 120.00) | 41.10 (5.00 - 124.00) | 0.93 |
| *HbF elevated for age, n (%)* | 2 (15.38%) | 11 (52.38%) | 0.10 |
| *Karyotype* |  |  |  |
| *abnormal, n (%)* | 4 (30.77%) | 7 (33.33%) | 0.96 |
| *Monosomy 7, n (%)* | 3 (23.08%) | 2 (9.52%) | 0.56 |
| *Secondary mutations at diagnosis, n (%)* | 3 (23.08%) | 7 (33.33%) | 0.52 |
