# Supplementary material for: Exploring the genetic and epigenetic origins of juvenile myelomonocytic leukemia using newborn screening samples
Source: Leukemia. 2021 Jun 28;36(1):279–82. doi: 10.1038/s41375-021-01331-0 (PMC8720242; doi:10.1038/s41375-021-01331-0)
Supplement: Supplementary file 6 — Supplemental Figure Legends [file 41375_2021_1331_MOESM6_ESM.docx]

***Supplemental Figures***

***Supplemental Figure 1:*** (A) Spectrum of primary mutations at diagnosis (absolute numbers shown). (B) Spectrum of primary mutations at birth. (C) Detectability of somatic alterations at birth vs at diagnosis (absolute numbers shown).

***Supplemental Figure 2:*** Outcome based on alteration status at birth: (A) Overall survival (%) and (B) Event-free-survival (%) from time of diagnosis (years). Note: Patients with germline mutations had LOH at diagnosis but not a birth and are thus included in the “somatic alteration absent at birth” cohort.

***Supplemental Figure 3*:** JMML and Control NBS beta values were filtered for minimum reads (>49) and 1386 CpG loci from the international consensus definition were used for unsupervised hierarchical clustering using Ward's method. Three samples (on the right) were the most dissimilar to other samples and all had an *NRAS* alteration.

***Supplemental Figure 4:*** Serial VAF and methylation profiling of UPN3153 shows that the VAF of the patient’s mutation (*KRAS* p.G12D) changes over time; however, methylation profiling can detect the IM signature even when the disease burden decreases.
