# Supplementary figures and images for: Exploring the genetic and epigenetic origins of juvenile myelomonocytic leukemia using newborn screening samples
Source: Leukemia. 2021 Jun 28;36(1):279–82. doi: 10.1038/s41375-021-01331-0 (PMC8720242; doi:10.1038/s41375-021-01331-0)

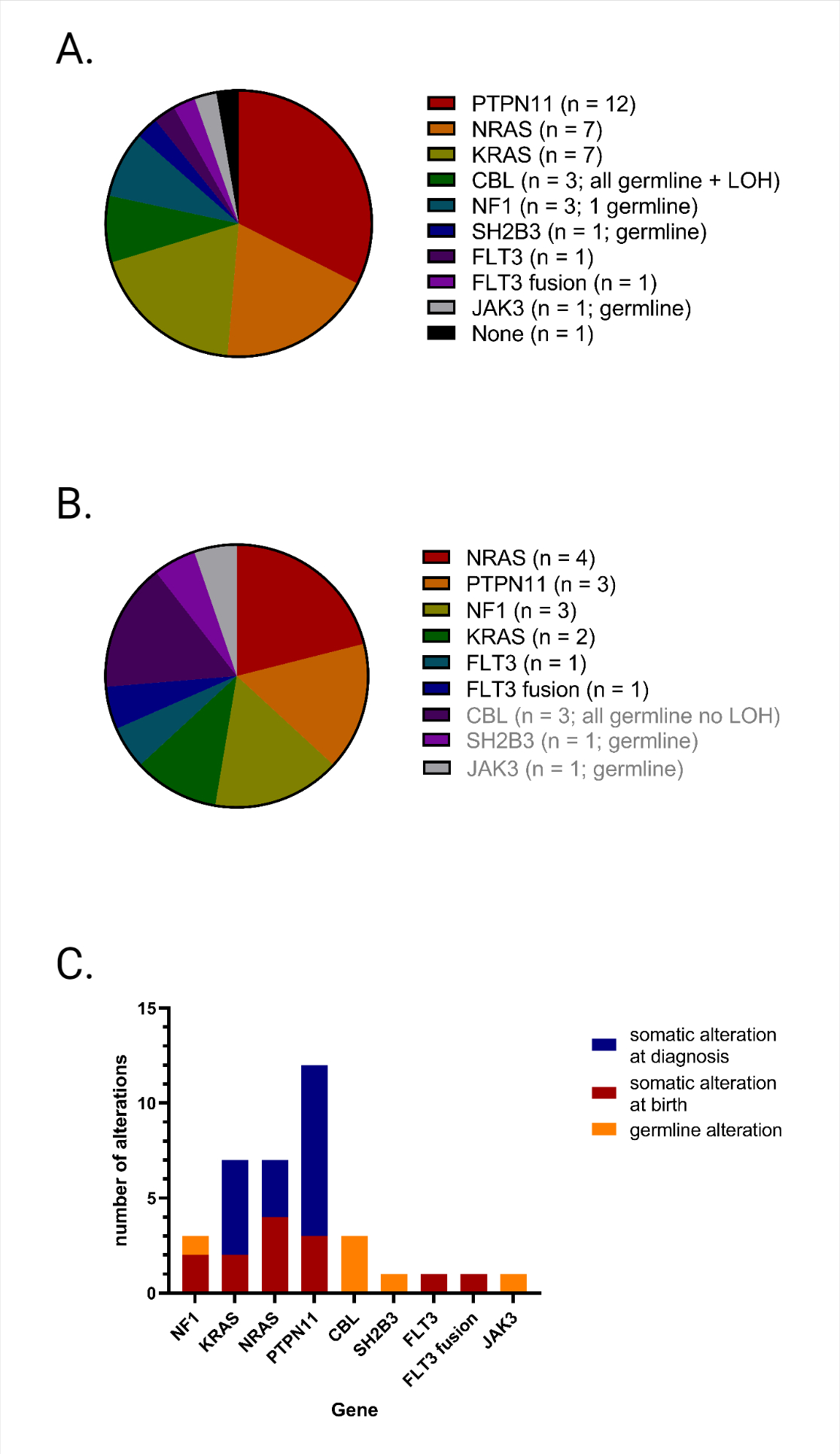

Supplement: Supplementary file 7 — Supplemental Figure 1 [file 41375_2021_1331_MOESM7_ESM.jpg]

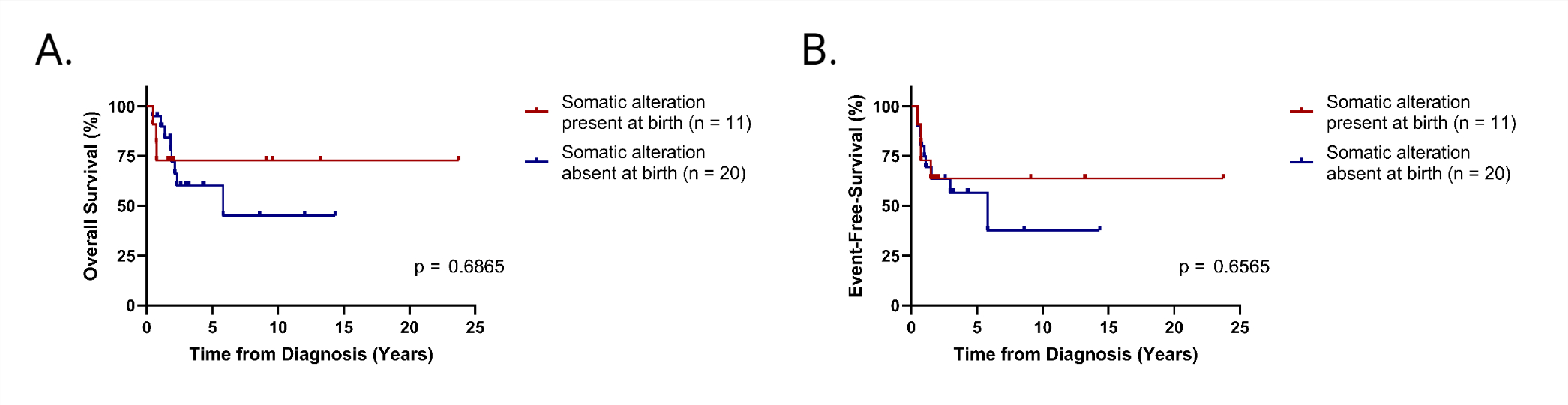

Supplement: Supplementary file 8 — Supplemental Figure 2 [file 41375_2021_1331_MOESM8_ESM.jpg]

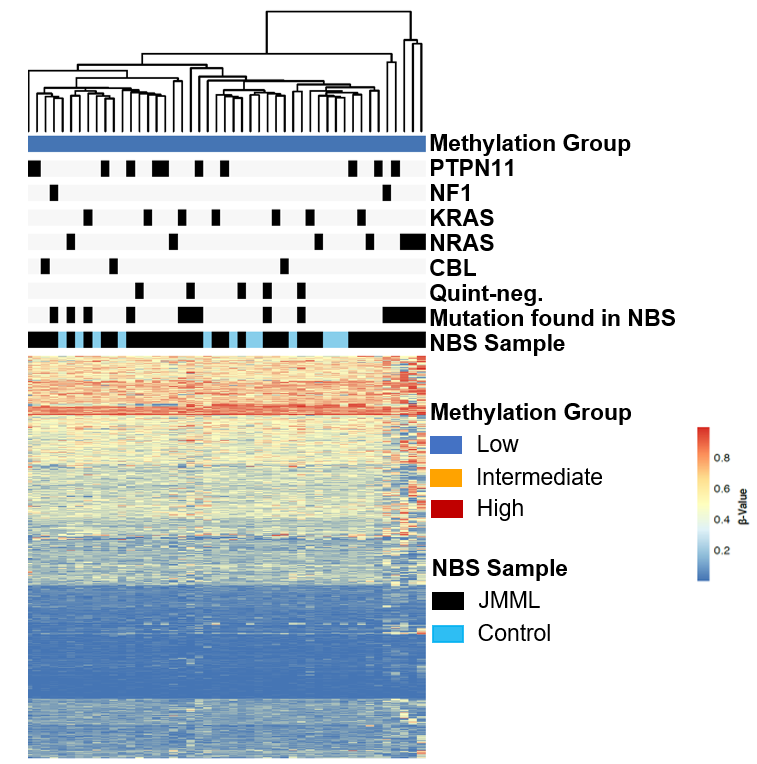

Supplement: Supplementary file 9 — Supplemental Figure 3 [file 41375_2021_1331_MOESM9_ESM.tif]

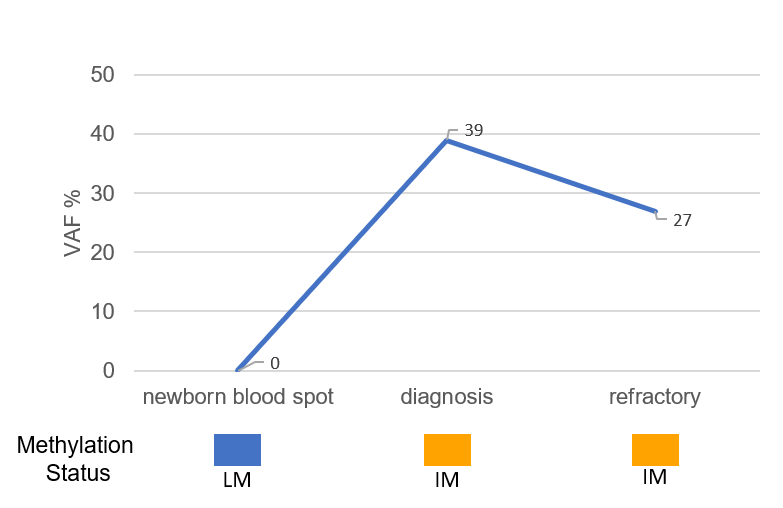

Supplement: Supplementary file 10 — Supplemental Figure 4 [file 41375_2021_1331_MOESM10_ESM.tif]
